# Rapid Detection of Microorganisms Based on Active and Passive Modes of QCM

**DOI:** 10.3390/s150100079

**Published:** 2014-12-23

**Authors:** Zdeněk Farka, David Kovář, Petr Skládal

**Affiliations:** 1 CEITEC MU, Masaryk University, Kamenice 5, 625 00 Brno, Czech Republic; E-Mails: farka@mail.muni.cz (Z.F.); kovar@nanobio.cz (D.K.); 2 Department of Biochemistry, Faculty of Science, Masaryk University, Kotlářská 2, 611 37 Brno, Czech Republic

**Keywords:** *Escherichia coli*, immunosensor, impedance analysis, label-free detection, quartz crystal microbalance

## Abstract

Label-free immunosensors are well suited for detection of microorganisms because of their fast response and reasonable sensitivity comparable to infection doses of common pathogens. Active (lever oscillator and frequency counter) and passive (impedance analyzer) modes of quartz crystal microbalance (QCM) were used and compared for rapid detection of three strains of *E. coli*. Different approaches for antibody immobilization were compared, the immobilization of reduced antibody using Sulfo‐SMCC was most effective achieving the limit of detection (LOD) 8 × 10^4^ CFU·mL^−1^ in 10 min. For the passive mode, software evaluating impedance characteristics in real-time was developed and used. Almost the same results were achieved using both active and passive modes confirming that the sensor properties are not limited by the frequency evaluation method but mainly by affinity of the antibody. Furthermore, reference measurements were done using surface plasmon resonance. Effect of condition of cells on signal was observed showing that cells ruptured by ultrasonication provided slightly higher signal changes than intact microbes.

## 1. Introduction

Rapid detection and identification of pathogenic bacteria is important in many fields of human activities: medicine, environment control, waste water treatment, food industry, *etc*. Microbiological methods require time consuming cultivation of bacteria, followed by biochemical or immunological tests. An effort exists to develop a rapid, sensitive and conclusive method for the detection of relevant pathogens. Immunosensors may help in this case, especially the label-free devices providing simplified assay formats. The two mostly used direct immunosensors are based on either optical surface plasmon resonance or mass sensitive quartz crystal microbalance (QCM) transducers.

QCM is a sensitive device consisting of the AT-cut quartz disc plate covered by evaporated electrodes (Au, Pt, *etc.*) on both faces allowing further immobilization of sensitive biorecognition elements. Alternating voltage applied to these electrodes induces shear deformation of the crystal. The Curie brothers discovered this phenomenon called piezoelectricity in 1880 [1]. The first sensor application was developed after Sauerbrey derived a formula for changes of the oscillation frequency depending on the mass loaded to the sensor surface [2]. The behavior of the crystal with respect to Sauerbrey's equation is effective in vacuum and also under special conditions in air. A significant step forward was also elucidation of the behavior of the QCM sensor in liquids [3,4].

Many QCM-based biosensors were developed and tested over almost 30 years [5–8]. Two basic techniques have been developed and specified in a theoretical comprehension for getting the accurate frequency changes. The first one inserts the crystal to an oscillator circuit with elements that are explicitly related to physical properties of the crystal. In this way, it is rather difficult to distinguish between mass changes and viscosity effects of liquid. This technique is known as active mode or classical QCM. The other approach requires more expensive impedance analyzer. The impedance characteristic is measured close to the resonant frequency of the crystal allowing to differentiate between mass-affected frequency change and the contribution of liquid [9,10]. Similarly, this technique is known as passive mode.

Direct label-free detection of bacteria or viruses in liquid samples is a challenging task. QCM sensors have been tested for the detection of bacteria, viruses, fungi and algae. A QCM sensor for the detection of *Salmonella typhimurium* was developed and used for either direct or sandwich detection with gold nanoparticles [11]. Probiotic bacteria were detected in real samples in the range 10^4^–10^5^ CFU·mL^−1^ within 60 min [12]. Non-labelled detection of *Francisella tularensis* was demonstrated by Kleo using QCM with dissipation monitoring and a detection limit of 4 × 10^3^ CFU·mL^−1^ in 20 min [13]. *In vitro* study of infective endocarditis was also realized using a QCM in a flow system [14]. In the all the above cases antibodies were utilized as the biorecognition part. Alternatively, some viruses can serve for bacterial recognition. The specific phage-bacteria interaction was used for discrimination of methicillin resistant (MRSA) and sensitive (MSSA) strains of *Staphylococcus aureus* [15].

Most QCM sensors operate at the fundamental frequency in the range of 5–20 MHz. In some cases it is possible to apply an overtone frequency. Sensor response to *S. aureus* was measured at the 3rd overtone of the 5 MHz crystal, at 15 MHz [16]. An oscillator designed to drive the quartz crystal at 27 MHz (3rd overtone) was used for detection of the toxic algae *Alexandrium minutum* [17]. The response was quite large (−540 Hz) for concentration of algae 5.6 × 10^6^ CFU·mL^−1^, nevertheless, LOD was only 10^6^ CFU·mL^−1^. The authors concluded that the sensor response in a gravimetric regime is not well respected. Beside the overtone oscillators, a high fundamental frequency 50 MHz QCM oscillator circuit was designed as a DNA biosensor [18].

The main limitations of label-free QCM immunosensors are rather high values of LOD. Two main approaches have been utilized for elimination of this disadvantage: a nanoparticles-based preconcentration and amplification. The QCM sensor has been described for detection of *S. typhimurium* with simultaneous measurements of the resonant frequency and motional resistance. Using magnetic beads preconcentration and amplification, the achieved LOD was at 100 CFU·mL^−1^ based on motional resistance changes [19]. A label-free capacitive QCM immunosensor was developed for detection of *E. coli* O157:H7 with LOD equal to 220 CFU·mL^−1^ within 1 h [20].

The theory of QCM detection of living microbial particles is still not completely clear. Mathematical models and descriptions of sensor behavior have been published [21]. One could expect a negative shift of frequency during an interaction of these particles with sensor. However, in some cases, a positive shift can occur and the sensors response is not as expected [22,23]. Besides transduction, affinity of the biorecognition part and method of its immobilization at the sensing surface play a significant role.

The available information indicates that passive mode is not routinely employed for detection of the living bacteria in flow liquids. Usually, small inorganic or biological molecules are tested and the detection is not carried out in flow systems [24]. This work describes a comparison of active and passive modes for determination of the resonant frequency corresponding to binding of bacteria to antibodies realized in a flow-through system. The specificity of the antibodies was tested on several strains of *E. coli*. SPR measurements were included to check the binding kinetics and the atomic force microscopy imaging was applied for confirmation of microbial cells in the sensing surface. Finally, the effect of bacterial state (viable, dead, desintegrated) on the responses was studied.

## 2. Experimental Section

### 2.1. Chemicals and Reagents

(3-Aminopropyl)triethoxysilane (APTES), cysteamine, glutaraldehyde (GA), staphylococcal protein A (SpA) and sulfosuccinimidyl-4-(*N*-maleimidomethyl)cyclohexane-1-carboxylate (Sulfo‐SMCC) for sensor surface modifications were purchased from Sigma-Aldrich (St. Louis, MO, USA). Chemicals for buffer preparation were obtained from PENTA (Prague, Czech Rep.). Phosphate buffered saline consisting of 50 mM sodium hydrogen phosphate/sodium dihydrogen phosphate and 150 mM sodium chloride pH 7.4 (PBS) was used for QCM analysis, PBS-EDTA (100 mM PBS, 10 mM EDTA, pH 7.2) was used for preparation of reduced antibodies. Acetate buffer (50 mM, pH 4.5) was prepared by mixing acetic acid and sodium acetate. For regeneration of sensor surface, either 50 mM sodium hydroxide or 100 mM citrate buffer pH 4.0 was used. Piranha solution was prepared by mixing concentrated sulfuric acid and 30% hydrogen peroxide in a 3:1 volume ratio. 1-Ethyl‐3-(3-dimethyl-aminopropyl)carbodiimide (EDC), *N*-hydroxysuccinimide (NHS), ethanolamine and HBS-P buffer for SPR experiments were purchased from GE Healthcare (Uppsala, Sweden). All solutions were filtered through a 0.22 μm PTFE membrane (Merck Millipore, Billerica, MA, USA).

### 2.2. Microorganisms and Antibodies

The used *E. coli* strains (BL21, DH5α and K-12) were obtained from the Czech Collection of Microorganisms and were all cultivated using the same procedure. Stock solution (100 μL) were inoculated into low salt LB Broth (200 mL, Duchefa Biochemie, Haarlem, The Netherlands) in Erlenmeyer flasks and the cultivation was done aerobically at 37 °C overnight. The obtained bacterial suspension was centrifuged thrice for 10 min at 4500 g and washed with sterile PBS. Concentration of bacteria was determined by measuring optical density at 600 nm, calibration was done by the McFarland scale.

Detection of the strains BL21 and DH5α was done using goat polyclonal antibody Abcam ab25823 (Abcam, Cambridge, UK). Rabbit polyclonal antibody Serotec 4329-4906 (AbD Serotec, Kidlington, UK) was used for detection of the strain K-12.

The capability of antibodies to bind *E. coli* cells was confirmed using atomic force microscopy (AFM). Glass cover slips were submerged in freshly prepared acidified methanol (methanol and chloric acid in volume ratio 1:1) for 30 min, washed with water and submerged in concentrated sulfuric acid for another 30 min [25]. After washing with water, their surface was activated with 2% APTES (in 95% methanol acidified with 2% HCl, pH 4.6) for 3 h at room temperature and in the dark. Then the activated slips were cured for 1 h at 110 °C and incubated with 5% glutaraldehyde for another 1 h. The antibodies (100 μg·mL^−1^) were immobilized directly to this layer overnight at 4 °C. Free reactive groups were deactivated using ethanolamine (50 mM, 30 min). The washed slips were stored in closed Falcon tubes.

Microbes (concentration 10^7^ CFU·mL^−1^) were allowed to bind for 1 h and then the surface was thoroughly washed with deionized water. The scanning was done in semicontact mode using the AFM NanoWizard 3 system (JPK Instruments, Berlin, Germany) and the ACTA-10 probe (Applied NanoStructures, Mountain View, CA, USA).

### 2.3. Preparation of Biosensing Layers for QCM

Three different approaches were used for immobilization of antibodies to gold electrodes of 10 MHz quartz crystals (ICM, Oklahoma City, OK, USA). The sensor surface was always initially cleaned for 30 min with chromic acid and rinsed with deionized water. In the first procedure, the surface was activated using cysteamine (20 mg·mL^−1^, 2 h at room temperature), glutaraldehyde (5%, 1 h at room temperature) and protein A (1 mg·mL^−1^, 20 h at 4 °C). In the next step, the antibody was bound (100 μg·mL^−1^, 20 h at 4 °C) and free reactive groups were deactivated using ethanolamine (50 mM, 30 min at room temperature). After each step, the sensor was thoroughly washed with sterile deionized water, allowed to dry and resonant frequency was measured using both frequency counter and impedance analyzer. The second approach was based on the same procedure but antibody was bound directly to glutaraldehyde omitting the protein A [26].

For the third method, antibody was diluted in PBS-EDTA to 2 mg·mL^−1^ and then it was reduced (10 μL of 60 mg·mL^−1^ cysteamine were added to 100 μL of antibody solution). The incubation was done for 90 min at 37 °C and the reduced antibodies were purified using centrifugal microfilter Microcon YM-10 (Merck Millipore, Billerica, MA, USA). The sensor surface was activated by cysteamine (20 mg·mL^−1^, 2 h at room temperature), Sulfo-SMCC (3 mg·mL^−1^, 1 h at room temperature) and finally, the reduced antibody was bound (100 μg·mL^−1^, 18 h at 4 °C). The third immobilization procedure leaves no free reactive groups and therefore no deactivation was required [27].

### 2.4. Active and Passive QCM Immunoassay

**Figure 1.**
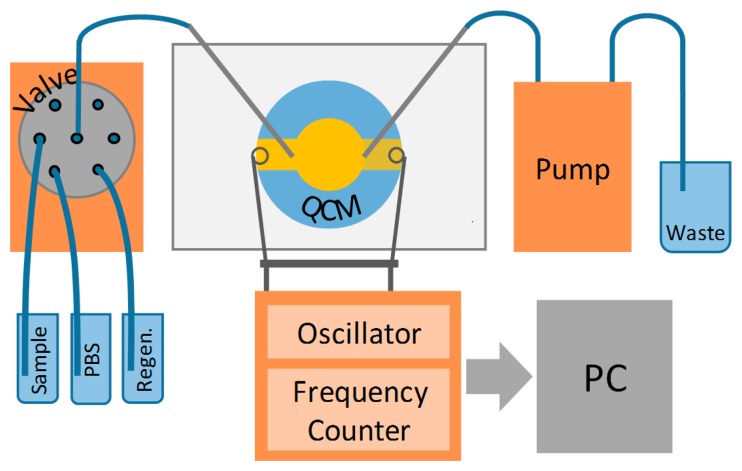
Scheme of experimental set-up for active QCM measurements.

Active QCM measurements were performed using the QCM Analyzer (KEVA, Brno, Czech Republic) which serves both as oscillator and frequency counter. Piezoelectric crystals with immobilized antibody were placed in a flow-through cell (designed and constructed by Karel Lacina). Transport of liquids was provided by a milliGAT pump (Global FIA, Fox Island, WA, USA) and a selection valve (Valco Instruments, Houston, TX, USA). A scheme of the experimental set-up is shown in Figure 1. The whole system was controlled via the in-house developed software LabTools that allowed fully automated operation. PBS was used as a running buffer with a flow-rate of 40 μL·min^−1^. After baseline establishing, samples were injected for 10 min followed by 10 min dissociation phase in PBS. Regeneration was done for 2 min using 50 mM NaOH in case of sensors with antibody bound using protein A and by citrate buffer in case of the other sensors.

Passive QCM measurements were done by an Agilent 4249A impedance analyzer (Agilent, Santa Clara, CA, USA) connected by a LAN network. The analyzer was continuously scanning the impedance characteristics near the crystal resonance frequency, and each spectrum consisted of 401 points and was measured with the parameter *Bandwidth 4* (the spectrum acquisition took 20 s). Resonant frequencies corresponding to the zero phase shift (*f*_r_) and maximum admittance (*f*_m_) were evaluated in real-time using LabTools software according to the built-in *OUTPRESO* fitting function. The amplitude of impedance at the resonant frequency (|*Z*_r_|) was obtained simultaneously. In contrast to the alternative BVD model (represented by the function *EQUCPARS4*), the frequency based on the zero phase shift provided more reliable results and lesser fluctuations for measurements in liquid. The remaining experimental parameters were the same as in case of the active QCM experiments.

### 2.5. SPR Immunoassay

Reference SPR measurements were performed on Biacore 3000 (GE Healthcare, Uppsala, Sweden) equipped with the CM5 chip. The Biacore microfluidic system contains four flow-through channels (Fc). Antibody immobilization was done in a flow-through mode (buffer HBS-P, flow-rate 5 μL·min^−1^). First, carboxymethylated dextran matrix was activated by 50 μL of 1:1 mixture of EDC (400 mM) and NHS (100 mM). Then, antibody (20 μL, 10 μg·mL^−1^, in acetate buffer pH 4.5) were injected into the channel Fc4. After this step the signal change was 5,000 RU. The channel Fc3 (serving as a reference) was modified by the same procedure but excluding antibody binding. The remaining reactive groups were deactivated using ethanolamine (30 μL, 1 M, pH 8).

For the measurements, the same experimental conditions were used (buffer HBS-P, flow-rate 5 μL·min^−1^). When the baseline signal was established, *E. coli* samples (50 μL) were injected using the *KINJECT* function. Ten min of association phase were followed by 10 min of dissociation in HBS-P, regeneration was done by a short pulse (10 μL) of 50 mM HCl. Differential signal (Fc4 − Fc3) was evaluated to suppress the effect of non-specific binding and refractory index changes.

## 3. Results and Discussion

### 3.1. Scanning by Atomic Force Microscopy

Atomic force microscopy was used to study microbial cells both nonspecifically attached to a cover slip glass and specifically bound to the antibody immobilized on glass. In the case of the nonspecific binding, the smoothness of the cover slip and shape of *E. coli* cells was checked.

**Figure 2.**
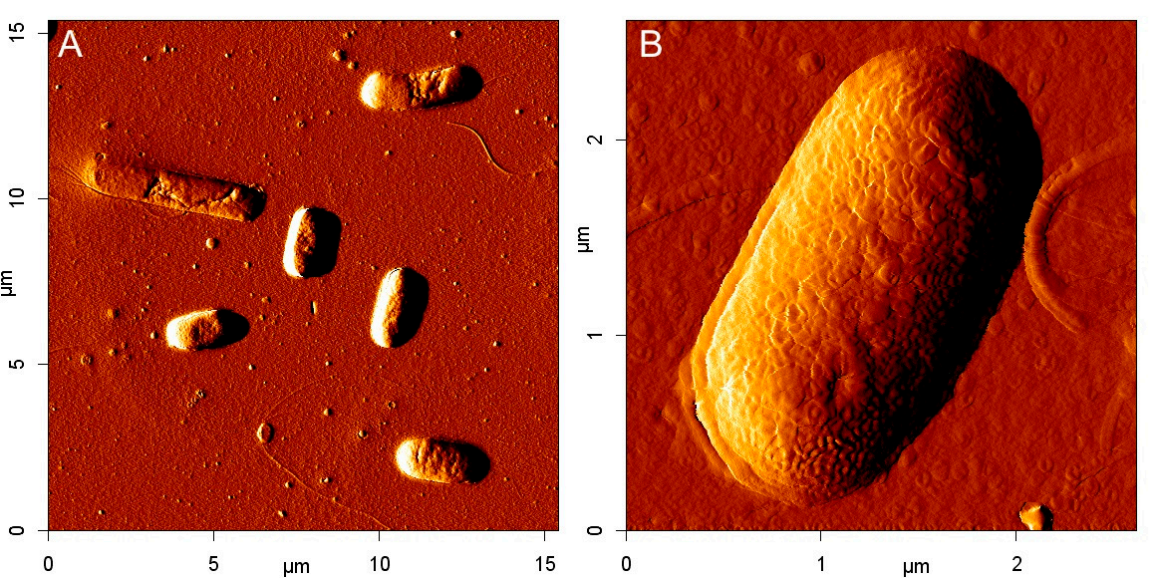
AFM scan of *E. coli* K-12 specifically bound on the cover slip modified by APTES and antibody Serotec 4329-4906. The “*Error*” signal is shown. (**A**) Overview and (**B**) detail of a single cell.

Compared to the practically flat bare cover slips, small nm-sized particles were formed on the APTES-modified slips. The Ab-modified glass slips were incubated with bacteria suspension (10^7^ CFU·mL^−1^) and after 1 h thoroughly washed with water. The samples were scanned in the semi-contact mode. High-density immobilization of antibodies led to high surface concentration of the bound bacteria (average 2 cells per 100 μm^2^) as evident in Figure 2. In the case of blank (glass slip modified using APTES, glutaraldehyde and ethanolamine, but with no antibody), no bacterial cells were captured. QCM sensor modified with reduced antibody was scanned, as well. In this case, the number of bound bacteria was smaller (0.5 cells per 100 μm^2^), but the antibody binding ability was still clearly confirmed.

### 3.2. Performance of the QCM Immunosensor

**Figure 3.**
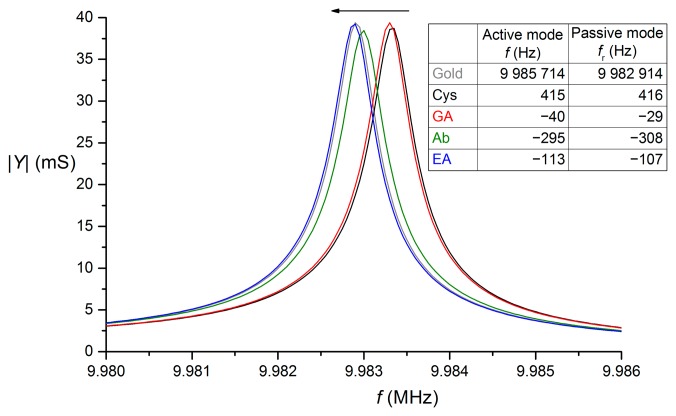
Impedance characteristics of quartz crystal after individual immobilization steps.

After each immobilization step, crystal resonant frequency and impedance characteristics (Figure 3) were measured in dry state. Two approaches were used to determine the resonant frequency from impedance characteristics—the first one (*f*_r_) was corresponding to zero phase shift and the second one (*f*_m_) to maximal admittance (minimal impedance). These two methods provided practically identical results. In the following text, passive mode results are expressed as frequency *f*_r_. During the immobilization, the frequency shifted to lower values keeping admittance size the same as for bare gold. After binding of cysteamine, a frequency increase was noticed. The etching mechanism of gold by alkanethiols was already proved by STM and AFM studies [28].

The inset table shows the initial resonant frequency and further frequency changes from the previous immobilization step evaluated using both active and passive modes. Cys—cysteamine; GA—glutaraldehyde; Ab—antibody; EA—ethanolamine.

Comparing the resonant frequencies obtained by the active and passive method, the absolute frequency values differ by nearly 3000 Hz. The variation results from the completely different approach for frequency determination, but the subsequent frequency changes are readily comparable. This technique can be used also for determination of microbe concentrations. However, due to the drying step, it is longer and more complicated than the on-line mode which is discussed later.

**Figure 4.**
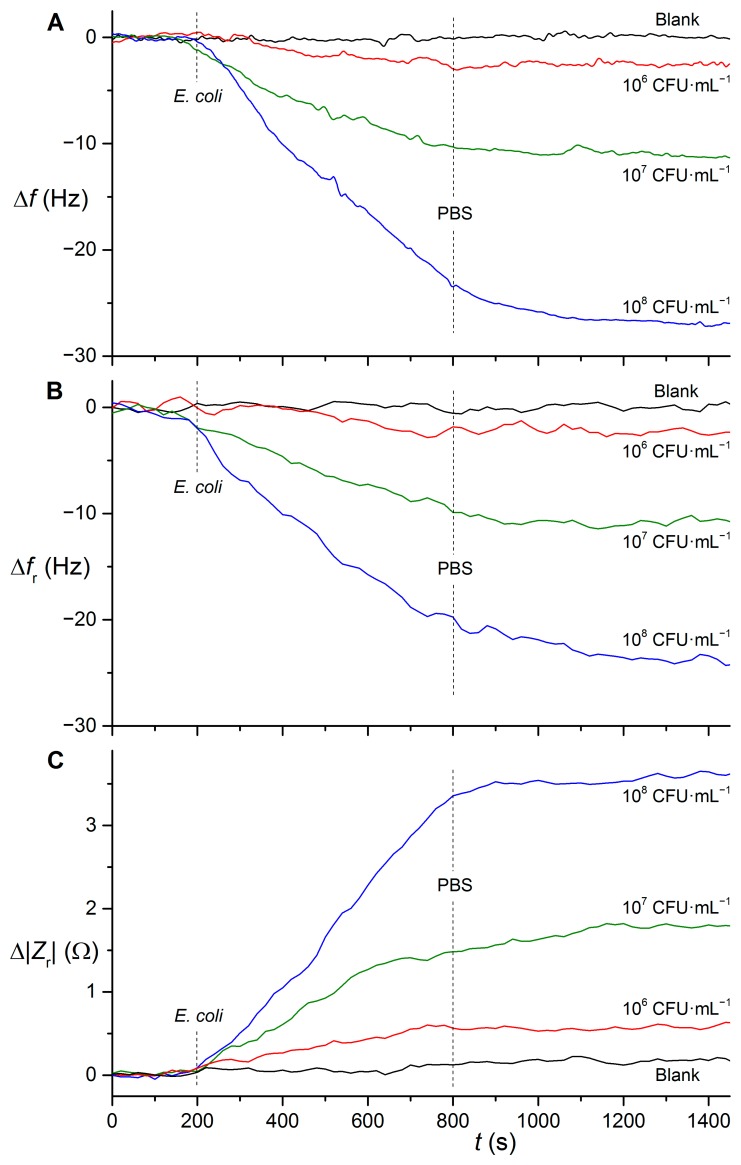
Binding interactions between *E. coli* DH5α and antibody Abcam ab25823 immobilized directly using glutaraldehyde. (**A**) Frequency measured in active mode using the QCM Analyzer; (**B**) Frequency measured in passive mode using Agilent 4294A; (**C**) Amplitude of impedance at the resonant frequency measured in passive mode.

The sensor with antibody Abcam ab25823 bound directly via glutaraldehyde was then used for detection of *E. coli* DH5α. The binding interactions were initially followed in the active mode (Figure 4A). The limits of detection (LOD) were determined as the *E. coli* concentration for which the signal reached three times of the standard deviation of blank measurement (3*s*). In this case the LOD was 9 × 10^5^ CFU·mL^−1^. Only 400 μL of sample was required for the experiments. The regeneration using citrate buffer pH 4.0 allowed reproducible detection of more than 15 samples with one side of the sensor. Practically no signal change was observed for either blank (PBS) measurements or cross-reactivity tests using 10^7^ CFU·mL^−1^ of *Bacillus atrophaeus* spores.

For the measurements using passive mode, all experimental conditions including microbe samples remained unchanged to suppress any effect other than the influence of frequency determination method. Binding interactions are shown in Figure 4B. The obtained LOD had the same value (9 × 10^5^ CFU·mL^−1^) as in case of active mode which confirms that both frequency determination methods are comparable. When evaluating the amplitude of impedance at the resonance, analogous results were achieved and the value of LOD was practically the same as using the frequency changes (8 × 10^5^ CFU·mL^−1^).

**Figure 5.**
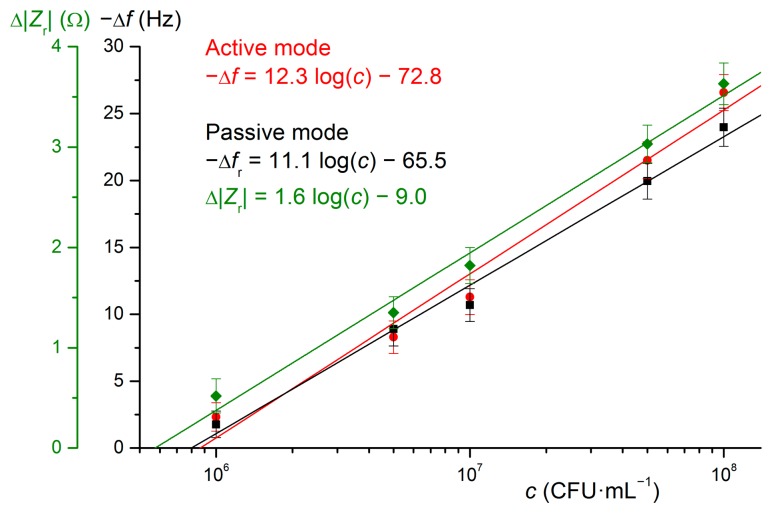
Calibration curves for determination of *E. coli* DH5α using active and passive QCM immunosensors based on antibody Abcam ab25823 immobilized directly using glutaraldehyde.

Figure 5 provides calibration curves for active and passive QCM detection of *E. coli* DH5α. Typically, the dependence of frequency change on concentration exhibits saturation character and therefore linearization by transforming *x* axis to log-scale is usually done. The sensor then provided a linear response up to 10^8^ CFU·mL^−1^. The error bars correspond to the standard deviations.

The other immobilization methods and *E. coli* strains were compared using the active mode. The results are summarized in Table 1. Calibration curves correspond only to the linear ranges of dependencies, LODs are evaluated using three times the standard deviation of blank signals. In all cases, the analysis time was only 10 min. The whole experiment including analysis, dissociation and regeneration phase took 35 min.

Antibody Abcam ab25823 immobilized using glutaraldehyde was used also for binding of *E. coli* BL21. In this case, the signal changes were approximately half of those for DH5α (slope 5.7 *vs.* 12.3 for DH5α) but LOD was similar (5 × 10^5^ CFU·mL^−1^). In a similar manner, the antibody Serotec 4329–4906 was immobilized via cysteamine and glutaraldehyde. Thus modified sensor was used for detection of K-12 strain. The LOD was the same as in the previous case. The slope was between values for both previously mentioned strains. Generally, these data suggest that lower LOD level probably cannot be achieved using this immobilization method.

Protein A provides oriented immobilization of antibodies because it binds their Fc fragment and therefore better LOD was expected. Even though the signals obtained for high concentrations of microbe were larger (slope 27.1), the sensor was not able to detect low *E. coli* concentrations. The higher value of LOD (5 × 10^6^ CFU·mL^−1^) was mainly due to the higher standard deviation of blank. In fact, this method provides oriented immobilization of antibody only relative to protein A, which can be bound also in a “nonproductive” way, despite its multivalency.

The best results were achieved with the reduced antibody bound via Sulfo-SMCC. Even though the signals for high concentrations were rather small (slope 5.1, which is comparable with the determination of the strain BL21), LOD of 8 × 10^4^ CFU·mL^−1^ was reached. Despite the fact this immobilization procedure is complicated (requiring reduced antibodies), it seems to be the most promising for sensitive detection of microorganisms using QCM.

Our findings confirm the previously published results, where conventional microbalances and electroacoustic admittance also gave the same response [17]. For the detection of microbes in food samples, lower LOD would be necessary. Further improvement of LOD can be achieved by various approaches—preconcentration using magnetic beads [29], cultivation based preconcentration [30] or by amplification using precipitation products [31,32]. Nevertheless, all of these methods would make the analysis times significantly longer.

### 3.3. Performance of the SPR Immunosensor

**Figure 6.**
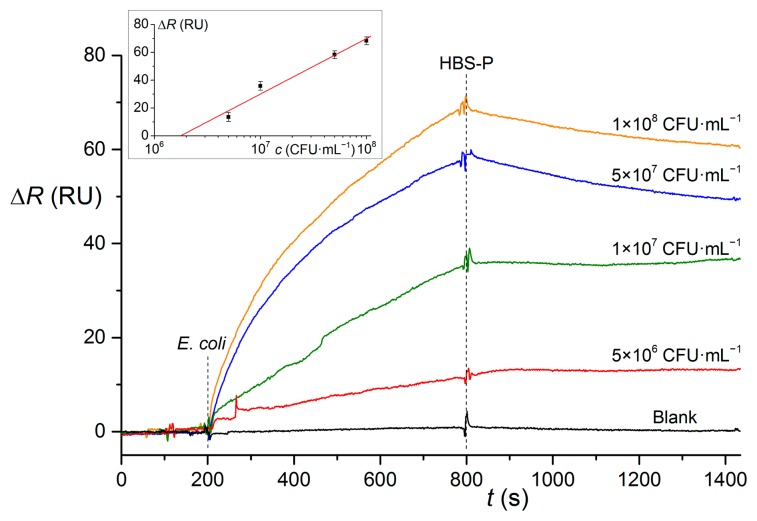
Binding interactions between *E. coli* DH5α and antibody Abcam ab25823 studied using surface plasmon resonance. Changes of differential signal are shown. The inset graph represents calibration curve for SPR detection of *E. coli* DH5α.

The binding interactions between *E. coli* DH5α and the antibody Abcam ab25823 were studied also using surface plasmon resonance (Figure 6). The SPR experiments were performed with 10 min of the binding phase as well. Regeneration was done by 50 mM HCl and this allowed more than 40 experiments with a single chip. The calibration curve and LOD (2 × 10^6^ CFU·mL^−1^, Table 1) are comparable to the results achieved using QCM. An improved LOD could be achieved using a dedicated LSPR system based on long-range surface plasmons combined with immunomagnetic entrapment of bacteria [33].

The producer claims that the Abcam ab25823 antibody is specific against heat-killed and sonicated *E. coli* cells, so both of these procedures were tested for expected improved response. First, the ultrasonication using a Sonopuls HD 2200 homogenizer with a MS 72 sonotrode (both from Bandelin Electronic, Berlin, Germany) was done. While the signal change for 10^8^ CFU·mL^−1^ of native cells was 68 RU, in case of ultrasonicated ones, the signal slightly increased to 87 RU. Typically, SPR recognizes only those parts of cells that are close to the sensor surface (in the range of surface plasmons); smaller cell pieces are detected entirely. Still, the difference was not too large and rough estimation of damaged cells could be done even using a common calibration curve. Heat-killing of cells was done in an autoclave at 121 °C for 20 min. For this sample, the signal change was only 12 RU, which indicates that thermal denaturation changed the antigenic structures and hindering binding to antibodies.

## 4. Conclusions

A piezoelectric immunosensor for rapid detection of microorganisms was developed. As a model microbe, *E. coli* strains BL21, DH5α and K-12 were used. From the three tested immobilization procedures, the best results were achieved with the reduced antibody bound via Sulfo-SMCC, and this sensor was able to detect 8 × 10^4^ CFU·mL^−1^ in 10 min, while regeneration allowed more than 15 measurements with one side of the QCM sensor. Active and passive QCM modes were compared; it seems that the effect of frequency determination method is negligible compared to the influence of the antibody immobilization procedure. The immunosensor shows good potential for detection of pathogenic microorganisms in common situations. Reference measurements were done using surface plasmon resonance; the ability of antibodies to bind *E. coli* cells was confirmed with AFM. The affinity of antibodies was found to be affected by cell viability and integrity. Still, the ability of the developed sensor to detect damaged cells was not substantially worse than in the case of living ones.

## Figures and Tables

**Table 1.** Summary of the results for tested QCM and SPR immunosensors and *E. coli* strains.

**Mode**	**Immobilization**	**Antibody**	**Strain**	**Calibration Curve**	**LOD (CFU·mL^−1^)**
**Active QCM**	Cys–GA	ab25823	DH5α	−Δ*f* = 12.3 log(*c*) − 72.8	9 × 10^5^
	Cys–GA	ab25823	BL21	−Δ*f* = 5.7 log(*c*) − 32.0	5 × 10^5^
	Cys–GA	4329-4906	K-12	−Δ*f* = 7.0 log(*c*) − 35.0	5 × 10^5^
	Cys–GA–SpA	ab25823	DH5α	−Δ*f* = 27.1 log(*c*) – 182	5 × 10^6^
	Cys–SMCC	4329-4906	K-12	−Δ*f* = 5.1 log(*c*) − 24.4	8 × 10^4^

**Passive QCM**	Cys–GA	ab25823	DH5α	−Δ*f* = 11.1 log(*c*) − 65.5	9 × 10^5^
				Δ|*Z*_r_| = 1.6 log (*c*) – 9.0	8 × 10^5^

**SPR**	EDC/NHS	ab25823	DH5α	Δ*R* = 39.9 log(*c*) – 249	2 × 10^6^

Cys—cysteamine, GA—glutaraldehyde, SpA—protein A, SMCC—Sulfo-SMCC.
